# Resveratrol in Rodent Models of Parkinson’s Disease: A Systematic Review of Experimental Studies

**DOI:** 10.3389/fphar.2021.644219

**Published:** 2021-04-22

**Authors:** Cheng-Fu Su, Li Jiang, Xiao-Wen Zhang, Ashok Iyaswamy, Min Li

**Affiliations:** ^1^ College of Pharmacy, Henan University of Chinese Medicine, Zhengzhou, China; ^2^ Mr. & Mrs. Ko Chi-Ming Centre for Parkinson’s Disease Research, School of Chinese Medicine, Hong Kong Baptist University, Hong Kong, China; ^3^ Department of Nutrition Science and Food Hygiene, Xiangya School of Public Health, Central South University, Changsha, China; ^4^ State Key Laboratory of Quality Research in Chinese Medicine, Institute of Chinese Medical Sciences, University of Macau, Macao SAR, China

**Keywords:** Parkinson’s disease, resveratrol, neuroprotective effects, PD animal models, meta-analysis

## Abstract

Parkinson’s disease (PD) is a common neurodegenerative disease featured by progressive degeneration of nigrostriatal dopaminergic neurons (DA) accompanied with motor function impairment. Accumulating evidence has demonstrated that natural compounds from herbs have potent anti-PD efficacy in PD models. Among those compounds, resveratrol, a polyphenol found in many common plants and fruits, is more effective against PD. Resveratrol has displayed a potent neuroprotective efficacy in several PD animal models. However, there is still no systematic analysis of the quality of methodological design of these studies, nor of their results. In this review, we retrieved and analyzed 18 studies describing the therapeutic effect of resveratrol on PD animal models. There are 5 main kinds of PD rodent models involved in the 18 articles, including chemical-induced (MPTP, rotenone, 6-OHDA, paraquat, and maneb) and transgenic PD models. The neuroprotective mechanisms of resveratrol were mainly concentrated on the antioxidation, anti-inflammation, ameliorating mitochondrial dysfunction, and motor function. We discussed the disadvantages of different PD animal models, and we used meta-analysis approach to evaluate the results of the selected studies and used SYRCLE’s risk of bias tool to evaluate the methodological quality. Our analytical approach minimized the bias of different studies. We have also summarized the pharmacological mechanisms of resveratrol on PD models as reported by the researchers. The results of this study support the notion that resveratrol has significant neuroprotective effects on different PD models quantified using qualitative and quantitative methods. The collective information in our review can guide researchers to further plan their future experiments without any hassle regarding preclinical and clinical studies. In addition, this collective assessment of animal studies can provide a qualitative analysis of different PD animal models, either to guide further testing of these models or to avoid unnecessary duplication in their future research.

## Introduction

Parkinson’s disease (PD) is a prevalent neurodegenerative disease with defective motor function. Around the globe, 2–3% of people aged over 65 suffer from PD. People with PD may have trouble in walking, talking, or doing simple tasks (Poewe et al., 2017). Disabilities of the motor system, including stiffness, bradykinesia, tremor, and unstable posture are the main syndromes of PD (Dickson, 2018; Homayoun, 2018). The major neuropathological marks of PD are neuronal loss and the accumulation of Lewy bodies (LBs). LBs consist of misfolded and aggregated α-synuclein which is involved in synaptic transportation (Wang et al., 2017). α-synuclein mainly aggregates in neurons, and current research has shown that its toxic conformations are protofibrils and oligomers (Lashuel et al., 2013). Moreover, mitochondrial deficits and neuroinflammation also appear to be related to the pathogenesis of PD (Greenamyre et al., 1999; Hirsch and Hunot, 2009).

Currently, strategies for treating PD can be classified into five types as follows: gene therapy, neuroprotective drugs, anti-inflammatory drugs, stem cells, and neurotrophic factors. Some of the methods (e.g., stem cells) are prohibitively expensive for normal people; and all have side effects to some degree. Therefore, there is a need of safe, effective, and affordable drugs that can be administered over the long term. Resveratrol (3,4,5-trihydroxy-*trans*-stilbene) is a natural polyphenol which was first isolated from the roots of *Veratrum grandiflorum* Loes (white hellebore). It was also extracted from the roots of *Polygonum cuspidatum* which was commonly used in traditional Chinese and Japanese medicine (Baur and Sinclair, 2006). Currently, resveratrol can be found in a variety of plants such as *P. quinquefolia* (L.) Planch, *Paeonia lactiflora*, and *Morus alba* (Chun-Fu et al., 2013), and in several common foods like berries, peanuts, red wine, and red grapes (Oliveira et al., 2017). A variety of experiments have indicated that resveratrol appears to have ameliorating effects on PD models (Okawara et al., 2007), and many types of research have been operated to look into these effects. However, many of the studies have methodological flaws that make their results and conclusions questionable. For example, some studies showed bias in their research design. Other studies are hard to compare because of methodological differences; for instance, several studies used the same toxin to induce the PD animal models, whereas with different administration times and dosages. Besides, different researchers proposed different mechanisms as to the neuroprotection of resveratrol (Guo et al., 2016; Huang et al., 2019). However, a sole research cannot elucidate all the details of the mechanisms of resveratrol on PD. Addressing all these problems, this review systematically analyzes the bias of each study, and then summarizes the mechanisms of resveratrol, and discusses the disadvantages of different PD animal models. And this review can provide a general description to different animal models of PD, either to guide further testing of the model or to avoid unnecessary duplication. We systematically analyzed these studies aiming to ameliorate the quality of PD animal study and support some helpful information for clinical studies of resveratrol.

## Methods

### Search Strategy

We searched the literature to find articles on the neuroprotective effects of resveratrol in animal PD models. We searched three data resources: “PubMed,” “Web of Science,” and “Google Scholar.” Our search designations were “[resveratrol (Title/Abstract)] AND [Parkinson disease (Title/Abstract) OR Parkinson’s disease (Title/Abstract)]”. The reviewers (Li Jiang and Cheng-fu Su) evaluated the accuracy of search results independently by reading the titles and abstracts of the identified studies according to the inclusion criteria, and 18 articles were selected for this review.

### Inclusion Criteria

(1) PD rodent models with no limitations as to species, gender, weight, or age were selected.
(2) The study included a control group with placebo and a resveratrol intervention group.
(3) The effectiveness of resveratrol on the PD rodent models was measured.

### Exclusion Criteria

(1) Review articles, replicated articles, and abstracts without full text.
(2) No measurement of the effect of resveratrol on PD animal models.
(3) Resveratrol was combined with other chemicals or drugs in the experiments on PD animal models.

### Quality Assessment

RevMan 5.3 (Cochrane Community, London, United Kingdom), ImageJ (Rawak Software Inc., Stuttgart, Germany), and SYRCLE’s risk of bias tool (Hooijmans et al., 2014) were used to analyze the data.

### Data Elicitation and Grade Evaluation

The specific data of these studies are provided below (Table 1). The information of Table 1 includes the following details: 1) author and year of publication; 2) rodent model, including species, gender, age/weight, and modeling methods; 3) resveratrol treatment, including medication dosage, route of administration, and duration of treatment; 4) outcome measures; and 5) pharmacological activity/mechanism. We did the meta-analysis for some parts of the results of the studies by forest plot with RevMan 5.3 (Cochrane Community, London, United Kingdom).

**TABLE 1:** Characteristics of included research articles.

Author (Year)	Rodent model	Resveratrol treatment	Outcome measurement (change with resveratrol: ↑ or ↓)	Pharmacological activity/mechanism
Blanchet et al. (2008)	Male C57BL/6 mice (3–4 weeks) injected with MPTP (i.p., 7 mg/kg or 10 mg/kg, 4 times at 2 h interval)	Dosage: 50 mg/kg/day; Ad: p.o.; administration time: 13 days; or Dosage: 50 mg/kg/day; Ad: p.o.; administration time: 20 days	High-performance liquid chromatography; HPLC (DOPAC, DA, HVA↑).Western blotting (WB), immunofluorescence (IF), and immunohistochemistry (IHC) (TH↑)	Increased the level of striatal tyrosine hydroxylase (TH)
Jin et al. (2008)	Sprague–Dawley (SD) rats (8–12 weeks) stereotaxic injected (right striatum) using 6-OHDA (5 µg)	Dosage: 10, 20 or 40 mg/kg/day; Ad: p.o.; administration time: 70 days	Rotational behavior testing, ultra microstructure analysis; RT-PCR (COX-2, TNF-α↓), WB(COX-2↓)	Alleviated mitochondrial tumefaction, decreased TNF-α mRNA level, and the expression of COX-2.
Lu et al. (2008)	Male Balb/C mice (20–25 g) injected using MPTP for 7 days (i.p., 30 mg/kg)	Dosage: 20 mg/kg/day; Ad: i.v.; administration time: 7 days	Rotarod trial, grasp strength analysis. Measurement of extracellular free Radicals (DHBA↓); histology evaluation	Alleviated neuronal loss by free radical scavenging
Anandhan et al. (2010)	Male C57BL/6 mice (30–35g) injected with MPTP for 4 days (i.p., 30 mg/kg)	Dosage: 50 mg/kg/day; Ad: p.o.; administration time: 4 days	Behavioral tests (rotarod trial, hang trial, narrow beam walking test), biochemical estimations (DOPAC↑, HVA↑, TBARS↓, GSH↑, GPx↑, SOD↓, CAT↓)	Reversed toxicity of MPTP through improving the dopamine and its metabolites levels, increasing the levels of GSH, GPx, and enhancing behavior performance
Khan et al. (2010)	Male Wistar rats (16 weeks) stereotaxic injected (right striatum) with 10 μg 6-OHDA	Dosage: 20 mg/kg/day; Ad: i.p.; administration time: 15 days	Behavioral tests (rotarod trial, apomorphine-induced circling behavior, stepping test), biochemical analysis (TBARS↓, GSH↑, GPx↑, GR↑, CAT↑, SOD↑, na+/k + -atpase); HPLC (DOPAC↑, DA↑); IHC (TH↑, COX-2↓)	Improved antioxidant status and alleviated dopamine loss
Wang et al. (2011)	Male Wistar rats (180–210 g) stereotaxic injected (right striatum) with 15 μg of 6-OHDA	Dosage: 20 mg/kg/day; Ad: p.o.; administration time: 14 days	Behavioral trial (rotarod trial); IHC (TH↑); reactive oxygen species (ROS↓); apoptosis↓	Increased the total antioxidant, resveratrol liposome played a better protection role than resveratrol
Mudo et al. (2012)	PGC-1α transgenic mice injected with 14 mg/kg MPTP 3 times within a time period of 3 h, then injected with 7 mg/kg MPTP for the fourth time	Dosage: 20 mg/kg/day; Ad: i.p.; administration time: 15 days	IHC (TH↑, DAT↑); WB (TH↑, SOD2↑); HPLC (DOPAC↑, DA↑); IP of PGC-1a↑	Increased PGC-1α gene transcription; triggered neuroprotection via SIRT1/PGC-1a
Srivastava et al. (2012)	Swiss albino mice (20–25 g) only intraperitoneally injected using paraquat (10 mg/kg; ip) or injected with paraquat (10 mg/kg; ip) combined with maneb (30 mg/kg; ip), two times a week, for 9 weeks	Dosage: 10 mg/kg/day; Ad: i.p.; administration time: 63 days	RT-PCR (Cyp2d22↑, VMAT-2↑); HPLC (DA↑); IHC (TH↑); WB (TNF-α↓, Bax↓, p53↑, P-p53↓, IL-1β↓, etc.)	Ameliorated Cyp2d22 expression and paraquat accumulation, enhanced neuroprotective effect
Lofrumento et al. (2014)	C57BL/6 mice (weighing 22–24 g) injected with MPTP (i.p., 20 mg/kg, 4 doses over 8 h period)	Dosage: 50 mg/kg/day; Ad: p.o.; administration time: 21 days	IHC (TH↑), RT-PCR (TH↑, IL-1b↓, SOCS-1↑, CD11b↓, TNF-a↓, etc.); WB (TH↑, IL-1b↓, IL-6↓, SOCS-1↑, CD11b↓etc.)	Increased DA neurons by ameliorating inflammatory reactions
Wang et al. (2015)	C57BL/6 mice (9–10 weeks) injected with the 20 mg/kg MPTP per 8 h for 21 days (i.p.)	Dosage: 50 mg/kg/day; Ad: p.o.; administration time: 21 days	RT-PCR (miR-214↑and α-synuclein↓); WB (α-synuclein↓); IHC (α-synuclein↓)	Reversed expression of miR-214 and of SNCA in MPTP PD mice model
Gaballah et al. (2016)	Wistar albino rats (200–250 g) injected with rotenone every other day for 21 days (s.c. 1.5 mg/kg)	Dosage: 20 mg/kg/day; Ad: p.o.; administration time: 21 days	Catalepsy test, rotarod test; the enzyme-linked immunosorbent assay (ELISA) (DA↑, caspase-3↓, IL-1β↓); DNA binding activity (Nrf-2↑)	Improved rotenone-induced ER stress by reducing the gene expression of CHOP and GRP78 and restrained caspase-3 level, inhibited xanthine oxidase activity; preserved intracellular oxidation balance by motivating Nrf2 signaling pathway
Guo et al. (2016)	Male C57BL/6 mice (24–28 g) injected with MPTP for five days (i.p., 30 mg/kg)	Dosage: 100 mg/kg/day; Ad: p.o.; administration time: 33 days.	Behavioral tests (open-field trial, stride length test, pole test). HPLC (DOPAC↑, DA↑, HVA↑).WB and IF (TH↑, SIRT1↑, LC3B↑, p62↓, etc.)	Increased TH and dopamine levels, ameliorated behavioral impairments; activated SIRT1; triggered autophagy to degrade α-synuclein
Zhao et al. (2017)	Male C57BL/6 mice (20–25 g, 10 weeks old) administered with rotenone for 28 days (p.o. 30 mg/kg)	Dosage: 50 mg/kg/day; Ad: p.o.,; Administration time: 35 days	Rotarod test; IHC (TH↑); WB(TH↑); HPLC (DA↑); iron staining	Ameliorated motor coordination, improved iron levels, elicited neuroprotective effect
Zhang et al. (2018)	Male A53T SNCA mice (12 months)	Dosage: 50 mg/kg/day; Ad: p.o.; administration time:50 days.	Behavioral test (open-field, pole test, hindlimb clasping test, object recognition test, Y-maze test); IHC (α-synuclein↓, TH↑, Iba-1↓); ELISA (TNF-α↓, IL-6↓etc.); WB(α-synuclein↓).	Decreased neuroinflammation and oxidative stress, ameliorated motor function and cognitive deficiency in the A53T SNCA mouse model
Palle and Neerati. (2018)	Wistar rats (180–250 g) injected with rotenone for 35 days (s.c. 2 mg/kg)	Dosage:40 mg/kg/day; Ad: p.o.; administration time: 35 days	Behavioral tests (rearing behavior, rotarod test); tricarboxylic acid cycle enzymes (citrate synthase, aconitase, succinate dehydrogenase); oxidative parameters (MDA↓, GSH↑); histopathology	Altered behavioral function, reduced oxidative stress, and improved mitochondrial dysfunction.
Huang et al. (2019)	SD rats (7 weeks) stereotaxic injected with 8 μg 6-OHDA (2 μg/μL) in a unilateral midbrain substantia nigra.	Dosage: 15 or 30 mg/kg/day; Ad: p.o.; administration time: 36 days	Behavioral tests (rotarod trial, open-field trial, grid test), WB (Bcl-2↑, Bax↓, pro-caspase-3↑, PI3K↑, p-Akt↑, etc.), IHC (TH↑)	Activated the PI3K/Akt signaling pathway
Liu et al. (2019)	Balb/c mice (10 weeks) injected with MPTP (i.p., 15 mg/kg for 7 consecutive days)	Dosage: 10 mg/kg/day; Ad: p.o.; administration time:7 days.	Behavioral tests (open-field trial, rearing test), IHC (TH↑), WB (TH↑, Akt↑, α-synuclein↓, cleaved-caspase-3↓; Bax↓, Bcl-2↑, IL-1β↓)	Improved motor dysfunction, increased Bcl-2 and pAkt/Akt ratio, reduced Bax and caspase-3 level, promoted dopamine neuron survival
Xia et al. (2019)	Male mice (11–12 weeks old) injected with four doses of 20 mg/kg MPTP at 8 h interims (i.p.)	Dosage: 50 mg/kg/day; Ad: p.o.; administration time: 21 days	RT-PCR (SNCA↓, MALAT1↓, and miR-129↑); luciferase assay; WB(α-synuclein↓)	Inhibited MALAT1 expression, modulated the MALATI/mir-129/SNCA signaling pathway

## Results

### Study Selection

**FIGURE 1:**
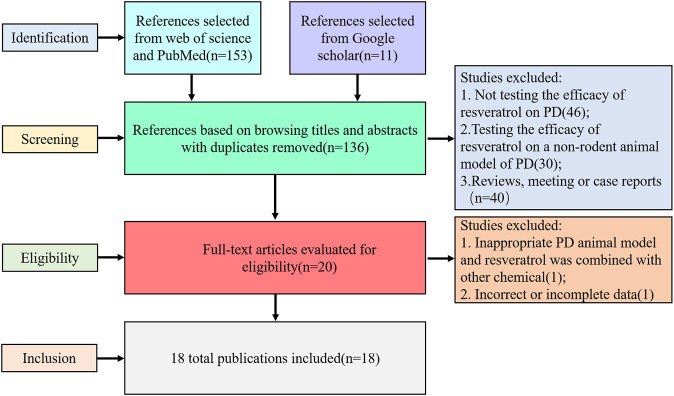
Selection methodology for study inclusion.

We identified 164 references, 153 from Web of Science and PubMed data resources, and 11 coming out of Google Scholar. Of these, 144 articles were excluded after browsing titles and abstracts. In the 144 excluded articles, 46 studies did not test the efficacy of resveratrol on PD; 30 articles tested the effect on a nonrodent animal model (cell model, zebra fish, and *Drosophila*); 40 were reviews, meeting or case reports; and 28 were duplicated publications. Through reading the remaining 20 articles, we found 1 study was based on a uncommon transgenic PD model and resveratrol was combined with another chemical in the treatment, and 1 study mainly focused on the nanoparticles of resveratrol; the data were incomplete, so we excluded the 2 studies. Therefore, 18 met our criteria (the inclusion criteria and exclusion criteria described in the method) and were used for meta-analysis (Figure 1). This result proved the efficiency of resveratrol on PD treatment and implied us that the neuroprotective mechanism of resveratrol was still worth exploring.

### Animal Models

In this review, 18 studies involved basically 2 kinds of PD rodent models: chemical-induced and transgenic animal models. Of the chemical-induced models, the 4 commonly used chemicals are as follows: 1-methyl-4-phenyl-1,2,3,6-tetrahydropyridine (MPTP); rotenone, 6-hydroxydopamine (6-OHDA), and paraquat (Tanner et al., 2011; Wang et al., 2020). We have listed the details of different models refereed to the 18 studies in Table 1. Here, we have summed up the different models in Table 2 to better know the pathological hallmarks, and the use in practical experiments of animal PD models. The MPTP model mainly mimics three aspects of the pathogenesis of PD: deprivation of dopaminergic neurons in the SNpc, defective mitochondrial respiration, and oxidative stress (Schober, 2004). When it passes through the blood-brain barrier (BBB), it is transformed into 1-methyl-4-phenylpyridinium ion (MPP^+^). This functioning metabolin is brought by dopamine shipper into DA of the SNpc where it harms the mitochondrial complex Ⅰ activity, causing L-DOPA–responsive Parkinsonian syndrome, with clinical features of PD (Heikkila et al., 1984; Liberatore et al., 1999; Blum et al., 2001). However, this kind of model does not lose neurons from locus coeruleus, a classical feature of PD (Dauer and Przedborski, 2003).

**TABLE 2:** Features of PD models.

Model	Mechanism	Behavior deterioration	Major usage of the model	Disadvantages
MPTP-induced	A reduction of striatal DA and TH	Dyspraxia	To generate irreversible and severe motor abnormalities	No loss of neurons from locus coeruleus; lack of age-dependent, slow progressive lesion development
Rotenone-induced	Suppresses mitochondrial complex I	Impaired motor activity	To trigger deterioration of nigrostriatal DA as well as aggregated proteins like α-synuclein	Age-independent lesions
6-OHDA-induced	Pro-oxidant properties, inhibits complex I activity	Rotational behavior	To induce motor impairment of limbs	Does not cross the BBB
Paraquat- and maneb-induced	Accelerates α-synuclein fibril formation	Impaired motor activity	To produce neuronal damage and a Parkinsonian-like syndrome	The dopaminergic toxicity is not selective
A53T transgenic	Produces mutations in SNCA	Motor deficits	Juvenile mice: usually without any overt phenotype; late middle-aged mice: development of dramatic motor phenotype	Expensive and time-consuming; the features of PD are not obvious

Rotenone is a tropical plant extract with cytotoxic effects. It can suppress mitochondrial complex I and trigger nigrostriatal DA degeneration and aggregated proteins like α-synuclein (Sherer et al., 2003; Liu et al., 2015). The 6-OHDA–induced model is the premier PD animal model; the neurotoxic effects of 6-OHDA are mainly caused by oxidative stress which is provoked by the formulation of superoxide, hydrogen peroxide, and hydroxyl radicals, as well as it can directly inhibit the complex I mitochondrial respiratory chain (Zhuang et al., 2020). However, it does not form LB-like inclusions like those observed in PD, and it cannot cross the BBB (Jonsson, 1980; Pycock et al., 1980).

Paraquat is a quaternary nitrogen herbicide, and it produces subcellular changes associated with PD (Widdowson et al., 1996). Paraquat can accelerate α-synuclein fibril formation *in vitro*. When systematically injected into mice, paraquat can significantly increase α-synuclein levels in the brain, particularly in the frontal cortex (Widdowson et al., 1996; McCormack et al., 2002). Oxidative stress and mitochondria impairment can also be triggered by paraquat (Berry et al., 2010).

Beyond the neurotoxin models, rodent transgenic models are also very helpful in PD study. SNCA mutations lead to unusual modalities of autosome-dominant PD; indeed, SNCA was coined to be linked with familial PD (Crabtree and Zhang, 2012). There are more than 30 known mutations (including E46K, A53T, and A30P) that have been recognized in SNCA gene (Meade et al., 2019). Different SNCA transgenic mice have been developed, in A53T transgenic mice, the A53T SNCA causes the formation of toxic fibrillar SNCA neuronal inclusions which can lead to motor function impairment. At the same time, the expression of the protein in catecholaminergic neurons reduces the expression of TH (Benskey et al., 2016).

Behavioral analysis motor function plays an important role in evaluating the drug’s effectiveness in PD. Most of the literatures listed here (12 of 18) investigated this function of animals. The two common methods to assess motor function are rotarod test and open-field test. The rotarod test coined by Miya and Dunham in 1957 is a tool to assess the effect of a medicine on animal behavior, especially neurological effects of a drug on rodents (Bohlen et al., 2009). It consists of a rod which is turning on a fixed axis with acceleration. For animals, the time they spend on it can reflect their motor coordination. Thus, an animal’s neuromuscular coordination can be assessed with the rotarod test (Shiotsuki et al., 2010). Nine research studies (50%) used the rotarod test to evaluate the protective effect of resveratrol.

Another test of coordination is the open-field test; it is popular and mainly used for rodents (Belzung, 1999). Usually, the field is marked with a grid and square intersection. The camera system can record the movement of animals, and the animals are chiefly depending on their tactual sense. Some determinants like the food or water deprivation can affect the exploration result (Prut and Belzung, 2003). In this review, 4 research studies (22%) performed the open-field test. Those articles revealed that resveratrol demonstrated a constant effect in ameliorating the motor functions in the behavioral experiments.

Nonmotor symptoms such as dementia, dysarthria, and hallucinations are also generally known in PD and may lead to notable disability (Lim and Lang, 2010). The object recognition test (ORT) is generally used to evaluate the memory and learning in rodent animals. In this test, the mice would be given 2 identical objects to observe whether they will spend more time on the novel object (Lueptow, 2017). The Y-maze test can be utilized to evaluate the short memory in mice (Kraeuter et al., 2019). In the selected literatures, one study performed the object recognition test and Y-maze test, and the results showed that resveratrol relieved cognitive deficiency in PD mouse model.

### Neuropathological Analysis

For better neuropathological analysis on PD models, we selected the form of forest plot by RevMan to meta-analyze the MPTP and 6-OHDA–induced PD models. Because only 5 studies showed the TH^+^ neuron counting numbers, we selected 3 studies which show TH^+^ neuron counting measured by IHC in MPTP-induced PD mice. A total of 15 MPTP-induced PD mice were treated with resveratrol via different doses and for different lengths of time (50–100 mg/kg/day, for 13–33 days), at the same time, the control group with 15 mice was administered with the vehicle.

**FIGURE 2:**
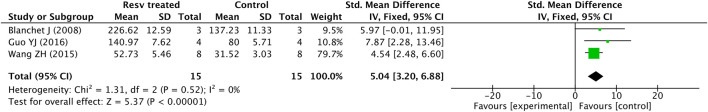
Forest plot for TH^+^ neuron counting measured by IHC in MPTP-induced PD mice: resveratrol vs. vehicle control. The mean difference and standard error of TH^+^ neuron counting in the resveratrol and vehicle-treated groups were quantificationally measured by ImageJ. These data were meta-analyzed in the form of forest plot by RevMan.

The meta-analysis outcome is displayed in the subsequent forest plot (Figure 2). The exploratory group was treated by resveratrol, the quantity of TH^+^ neurons was distinctly upregulated when it was compared with the vehicle-administered group (*p* < 0.00001), with an average difference of 5.04. Furthermore, there was no heterogeneity in the outcome (*p* = 0.52, I^2^ = 0%).

In 2 studies, a total of fourteen 6-OHDA–induced PD rats received different doses of resveratrol for different periods (4 SD rats with 15 mg/kg/day for 36 days and 10 Wistar rats with 20 mg/kg/day for 14 days), and 14 rats were relatively administered with vehicles.

**FIGURE 3:**
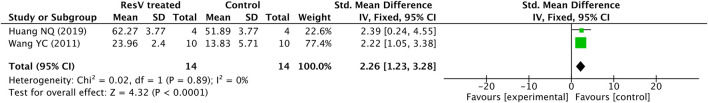
Forest plot for TH^+^ neuron counting measured by IHC in 6-OHDA–induced PD rats: resveratrol vs. vehicle control. The mean difference and standard error of TH^+^ neuron counting in the resveratrol and vehicle-treated groups were quantificationally measured by ImageJ. These data were meta-analyzed in the form of forest plot by RevMan.

The forest plot outcome is shown in Figure 3, the quantity of TH^+^ neurons were obviously increased in the resveratrol-treated group (*p* < 0.0001) in contrast with the vehicle group, with an average difference of 2.26. In addition, there was no heterogeneity in the total result (*p* = 0.89, I^2^ = 0%).

### Neuroprotective Mechanisms Analysis

**FIGURE 4:**
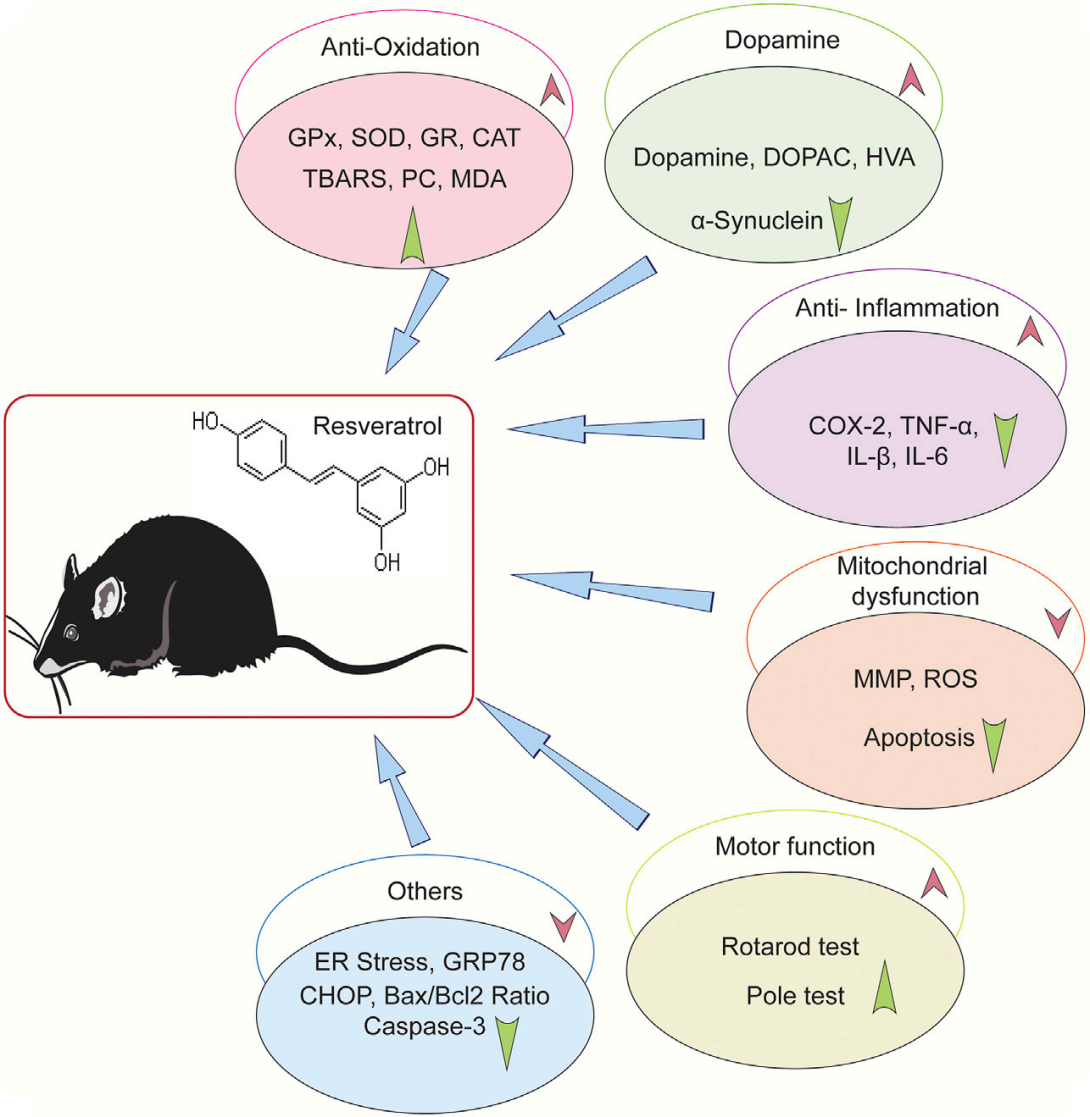
Neuroprotective mechanisms of resveratrol in animal PD model. “↑” Means upregulation, “↓” means down-regulation.

As is shown in Figure 4, a range of neuroprotective mechanisms for resveratrol were described in the selected studies. Most of the studies found that resveratrol ameliorated motor dysfunction, increased the level of dopamine and its metabolites, improved striatal TH protein levels, reduced the expression of α-synuclein, and improved the antioxidant status. Moreover, some studies illustrated that resveratrol reduced the neuroinflammatory reactions and regulated mitochondrial dysfunction.

Five studies reported the antioxidant effect of resveratrol. Anandhan et al. (2010) indicated that resveratrol improved the antioxidant function, enhanced the activity of glutathione (GSH) and glutathione peroxidase (GPx), as well as decreased superoxide dismutase (SOD) and catalase (CAT) levels. Khan et al. (2010) demonstrated that resveratrol enhanced the levels of glutathione reductase (GR), antioxidant enzymes (GPx, SOD, CAT), and protein carbonyl (PC). Its antioxidative activeness is the mechanism for its neuroprotective effects. Wang et al. (2011) indicated that resveratrol had antioxidant and radical scavenging ability, which resulted the protection of DA in PD rats. Mudo et al. (2012) showed that resveratrol elevated the antioxidants via increasing SOD2 and Trx2 levels in transgenic mice model. Palle et al. reported that resveratrol attenuated oxidative stress in rotenone-induced PD rat model.

Five literatures showed that resveratrol executed an anti-inflammatory function on the PD modes. Srivastava et al. (2012) indicated that resveratrol reduced microglial activation and neuroinflammation via increasing Cyp2d22 expression. Lofrumento et al. (2014) demonstrated that resveratrol sharply decreased glial activation, reduced TNF-αand IL-1β levels, enhanced TH-immunoreactivity, and increased the suppression of cytokine signaling-1 (SOCS-1). The mechanism may be related with its anti-inflammatory action by SOCS-1 induction (Lofrumento et al., 2014). Gaballah et al. (2016) and Zhang et al. (2018) showed that resveratrol ameliorated neuroinflammatory reaction. Liu et al. (2019) indicated that resveratrol decreased the α-synuclein in the striatum, increased the Bcl-2 level, reduced the caspase-3, Bax and IL-β levels, and improved the pAkt/Akt ratio. These effects enhanced striatum dopamine neuron survival.

Two studies show that resveratrol ameliorated mitochondrial dysfunction. Jin et al. (2008) indicated that resveratrol alleviated chromatin condensation and mitochondrial dysfunction, and it had neuroprotective effects related to anti-inflammation by declining COX-2 and TNF-α levels. Mudo et al. (2012) used a transgenic animal model to show that peroxisome proliferator-activated receptor-gamma coactivator-1α (PGC-1α) has a crucial role in oxidating stress and mitochondrial activity. The result showed that resveratrol had the same effect with overexpressing PGC-1α on protecting DA withstanding the MPTP-induced cell death. *In vitro* studies showed that resveratrol activated PGC-1α through deacetylating SIRT1, and increased SOD2 and Trx2 levels. Resveratrol played a neuroprotective role through the SIRT1/PGC-1α pathway.

Besides, Gaballah et al. (2016) reported resveratrol reduced ER stress through decreasing GRP78 and CHOP expression and inhibited the activity of caspase-3 in rat brain. Resveratrol also maintained the antioxidant status in the cell through activating the glutathione peroxidase and Nrf2 signaling pathway. Huang et al. (2019) reported that resveratrol reduced the 6-OHDA–caused apoptosis in Sprague–Dawley (SD) rats by motivating the phosphoinositide 3 kinase (PI3K)/protein kinase B (Akt) pathway and decreasing the Bax/Bcl-2 ratio and caspase-3 expression (Huang et al., 2019).

### Methodological Quality Assessment

It is important to use the risk of bias as an evaluation index to evaluate the methodological quality in the experiment design and interpretation of results. Here, we employed the SYRCLE’S risk of bias tool to assess the technical value of 18 studies as stated in the guidance of Hooijmans et al., 2014 In Table 3, “+” means a minor extent of bias in the mark assessment and “−” represents a big chance of bias. “※” means the study did not include enough data to assess the degree of bias.

**TABLE 3:** Methodological quality of studies.

Study	1	2	3	4	5	6	7	8	9	10	Score
Blanchet et al. (2008)	※	+	※	※	※	+	※	※	+	※	3
Jin et al. (2008)	※	+	※	+	※	+	※	−	+	※	4
Lu et al. (2008)	+	+	※	※	※	+	※	−	+	※	4
Anandhan et al. (2010)	※	+	※	+	※	※	※	−	+	※	3
Khan et al. (2010)	※	+	※	+	※	+	※	※	+	+	5
Wang et al. (2011)	+	+	※	※	※	+	※	※	+	+	5
Mudo et al. (2012)	※	+	※	※	※	+	※	※	+	※	3
Srivastava et al. (2012)	※	+	※	※	※	+	※	−	+	※	3
Lofrumento et al. (2014)	+	+	※	+	※	+	※	−	+	+	6
Wang et al. (2015)	※	+	※	※	※	※	※	−	+	※	2
Gaballah et al. (2016)	+	+	※	+	※	※	※	−	+	※	4
Guo et al. (2016)	+	+	※	+	※	+	※	−	+	※	5
Zhao et al. (2017)	+	+	※	+	※	+	※	−	+	※	5
Zhang et al. (2018)	※	※	※	+	※	+	※	−	+	※	3
Palle and Neerati, 2018	+	+	※	+	※	+	※	−	+	※	5
Huang et al. (2019)	+	+	※	+	※	+	※	※	+	※	5
Liu et al. (2019)	※	+	※	※	※	+	※	−	+	※	3
Xia et al. (2019)	※	+	※	※	※	+	※	※	+	※	3

1-stochastic distribution sequence; 2-analogous baseline traits; 3-distribution concealment; 4-stochastic housing; 5-blinded intervening; 6-random collection for outcome measurement; 7-blinded evaluation of result; 8-unfinished outcome data; 9-selecting outcome recording; 10-else sources of bias. +: yes; −: no; ※: unclear.

As shown in Table 3, in evaluating study quality on a scale of 1–10, the high score represents the high quality of the methodology in the articles. Most of the studies scored 2–6 in our validation. “Random allocation” was reported by 8 publications (44.4%); almost all the publications reported the “similar baseline features” (17 publications, 94.4%); for the “allocation concealment” and “blinded assessment of outcome,” there was no publication reported the two points; in addition, all the articles were “selective outcome reporting”; there were 15 articles (83.3%) that reported “random selection for outcome assessment”; 3 publications had other bias like analysis bias. Besides, sample size counting was not told in all the 18 articles; for animal experiment, sample size should be large enough to show a reasonable statistical significance yet small enough to avoid unnecessary use of animals.

## Discussion

Recently, a variety of natural compounds have been researched for their potentials to treat neurodegenerative diseases (Iyaswamy et al., 2020a; Iyaswamy et al., 2020b). Resveratrol is a small molecule which can be found in a range of common fruits and plants. Various studies show that resveratrol has neuroprotective effect (Jin et al., 2008; Lu et al., 2008; Della-Morte et al., 2009; Wang et al., 2015). The question is whether the results of these research studies are consistent and reliable.

The meta-analysis in this review employed an effective method to minimize the deviation of every study, which is very useful to assess the compound’s effect in preclinical research. In this systematic review, we systematically evaluated the neuroprotective effect of resveratrol in various PD models to provide effective evidence for further application of resveratrol as an alternative medicine in PD clinical treatment.

In this review, we meta-analyzed the outcome data of TH^+^ neuron counts of PD animal models by forest blot. The result showed that in the resveratrol administered group, the quantity of TH^+^ neurons was distinctly upregulated when it was in contrast with the group treated with vehicle in MPTP-induced PD mice. Also, the quantity of TH^+^ neurons was remarkably increased in the resveratrol administered group in contrast with the vehicle group in 6-OHDA–induced models.

This review assessed 18 published articles studying the effects of resveratrol on PD rodent models. Of the total 18 studies, 9 studies used MPTP-induced PD rodent models, 4 studies applied the 6-OHDA–lesioned PD models, 3 studies applied rotenone-induced PD models, 1 study used an A53T transgenic PD model, and 1 study used paraquat-induced PD models. In general, the studies showed that the protective mechanism of resveratrol is mainly involved in reducing the levels of α-synuclein and increasing TH protein levels. Some studies reported the changes in neuroinflammation, autophagy, and oxidative stress; a variety of signaling pathways, such as SIRT1/PGC-1α, PI3K/Akt, and MALATI/mir-129/SNCA, were also revealed. And for the 9 articles of MPTP-induced PD models, the neuroprotective effects of resveratrol were mainly due to antioxidation (3 articles) and anti-neuroinflammation (2 articles).

In summary, we systematically reviewed 18 studies evaluating the protective effectiveness of resveratrol in PD animal models, which were carefully screened from 3 databases. We have presented the effects of resveratrol and have discussed the mechanisms of action. The results of this study showed that resveratrol has obvious neuroprotective effects on different PD models via quantitative methods. The collective result in our review can supply useful information for researchers to further plan their future experiments. In addition, this collective evaluation of animal studies can supply a qualitative analysis of different PD animal models, either to guide further assessment of these models or to avoid needless duplication in their future research. Nevertheless, we feel that the meta-analysis outcomes supply sufficient proof for the ameliorative effect of resveratrol on PD animals to warrant further preclinical and clinical studies.

## Abbreviations

6-OHDA, 6-hydroxydopamine; BBB, blood-brain barrier; CAT, catalase; DA, dopaminergic neurons; ELISA, enzyme-linked immunosorbent assay; GPx, glutathione peroxidase; GR, glutathione reductase; GSH, glutathione; HPLC, high-performance liquid chromatography; IF, immunofluorescence; IHC, immunohistochemistry; LB, Lewy bodies; MPP^+^, 1-methyl-4-phenylpyridinium ion; MPTP, 1-methyl-4-phenyl-1,2,3,6-tetrahydropyridine; PC, protein carbonyl; PD, Parkinson disease; SD, Sprague–Dawley; SIRT1, sirtuin 1; SNpc, substantia nigra pars compacta; SOD, superoxide dismutase; SOCS-1, suppression of cytokine signaling-1; TH, tyrosine hydroxylase; WB, Western blotting.

## References

[B1] Anandhan A. Tamilselvam K. Vijayraja D. Ashokkumar N. Rajasankar S. Manivasagam T. (2010). Resveratrol Attenuates Oxidative Stress and Improves Behaviour in 1-Methyl-4-Phenyl-1, 2, 3, 6-tetrahydropyridine (MPTP) Challenged mice. Ann. neurosciences 17, 113. 10.5214/ans.0972-7531.1017304 PMC4116990 25205886

[B2] Baur J. A. Sinclair D. A. (2006). Therapeutic Potential of Resveratrol: the In Vivo evidence. Nat. Rev. Drug Discov. 5, 493–506. 10.1038/nrd2060 16732220

[B3] Belzung C. (1999). Measuring Rodent Exploratory behavior. Techniques in the Behavioral and Neural Sciences. Elsevier 13, 738–749. 10.1016/S0921-0709(99)80057-1

[B4] Benskey M. J. Perez R. G. Manfredsson F. P. (2016). The Contribution of Alpha Synuclein to Neuronal Survival and Function - Implications for Parkinson's disease. J. Neurochem. 137, 331–359. 10.1111/jnc.13570 26852372 PMC5021132

[B5] Berry C. La Vecchia C. Nicotera P. (2010). Paraquat and Parkinson's disease. Cell Death Differ 17, 1115–1125. 10.1038/cdd.2009.217 20094060

[B6] Blanchet J. Longpré F. Bureau G. Morissette M. Dipaolo T. Bronchti G. (2008). Resveratrol, a Red Wine Polyphenol, Protects Dopaminergic Neurons in MPTP-Treated mice. Prog. Neuro-Psychopharmacology Biol. Psychiatry 32, 1243–1250. 10.1016/j.pnpbp.2008.03.024 18471948

[B7] Blum D. Torch S. Lambeng N. Nissou M.-F. Benabid A.-L. Sadoul R. (2001). Molecular Pathways Involved in the Neurotoxicity of 6-OHDA, Dopamine and MPTP: Contribution to the Apoptotic Theory in Parkinson's disease. Prog. Neurobiol. 65, 135–172. 10.1016/s0301-0082(01)00003-x 11403877

[B8] Bohlen M. Cameron A. Metten P. Crabbe J. C. Wahlsten D. (2009). Calibration of Rotational Acceleration for the Rotarod Test of Rodent Motor coordination. J. Neurosci. Methods 178, 10–14. 10.1016/j.jneumeth.2008.11.001 19041892 PMC4380177

[B9] Chun-Fu W. Jing-Yu Y. Fang W. Xiao-Xiao W. (2013). Resveratrol: Botanical Origin, Pharmacological Activity and applications. Chin. J. Nat. Medicines. 11, 1–15. 10.1016/S1875-5364(13)60001-1

[B10] Crabtree D. M. Zhang J. (2012). Genetically Engineered Mouse Models of Parkinson's disease. Brain Res. Bull. 88, 13–32. 10.1016/j.brainresbull.2011.07.019 21839151 PMC3244549

[B11] Dauer W. Przedborski S. (2003). Parkinson's disease. Neuron. 39, 889–909. 10.1016/s0896-6273(03)00568-3 12971891

[B12] Della-Morte D. Dave K. R. Defazio R. A. Bao Y. C. Raval A. P. Perez-Pinzon M. A. (2009). Resveratrol Pretreatment Protects Rat Brain from Cerebral Ischemic Damage via a Sirtuin 1-uncoupling Protein 2 pathway. Neuroscience 159, 993–1002. 10.1016/j.neuroscience.2009.01.017 19356683 PMC2668125

[B13] Dickson D. W. (2018). Neuropathology of Parkinson disease. Parkinsonism Relat. Disord. 46, S30–S33. 10.1016/j.parkreldis.2017.07.033 28780180 PMC5718208

[B14] Gaballah H. H. Zakaria S. S. Elbatsh M. M. Tahoon N. M. (2016). Modulatory Effects of Resveratrol on Endoplasmic Reticulum Stress-Associated Apoptosis and Oxido-Inflammatory Markers in a Rat Model of Rotenone-Induced Parkinson's disease. Chemico-biological interactions 251, 10–16. 10.1016/j.cbi.2016.03.023 27016191

[B15] Greenamyre J. T. Mackenzie G. Peng T. I. Stephans S. E. (1999). Mitochondrial Dysfunction in Parkinson's disease. Biochem. Soc. Symp. 66, 85–97. 10.1042/bss0660085 10989660

[B16] Guo Y.-J. Dong S.-Y. Cui X.-X. Feng Y. Liu T. Yin M. (2016). Resveratrol Alleviates MPTP-Induced Motor Impairments and Pathological Changes by Autophagic Degradation of α-synuclein via SIRT1-Deacetylated LC3. Mol. Nutr. Food Res. 60, 2161–2175. 10.1002/mnfr.201600111 27296520 PMC6089356

[B17] Heikkila R. E. Manzino L. Cabbat F. S. Duvoisin R. C. (1984). Protection against the Dopaminergic Neurotoxicity of 1-Methyl-4-Phenyl-1,2,5,6-Tetrahydropyridine by Monoamine Oxidase inhibitors. Nature 311, 467–469. 10.1038/311467a0 6332989

[B18] Hirsch E. C. Hunot S. (2009). Neuroinflammation in Parkinson's Disease: a Target for neuroprotection? Lancet Neurol. 8, 382–397. 10.1016/s1474-4422(09)70062-6 19296921

[B19] Homayoun H. (2018). Parkinson disease. Ann. Intern. Med. 169, ITC33–ITC48. 10.7326/aitc201809040 30178019

[B20] Hooijmans C. R. Rovers M. M. De Vries R. B. Leenaars M. Ritskes-Hoitinga M. Langendam M. W. (2014). SYRCLE’s Risk of Bias Tool for Animal studies. BMC Med. Res. Methodol. 14, 1–9. 10.1186/1471-2288-14-43 24667063 PMC4230647

[B21] Huang N. Zhang Y. Chen M. Jin H. Nie J. Luo Y. (2019). Resveratrol Delays 6-Hydroxydopamine-Induced Apoptosis by Activating the PI3K/Akt Signaling pathway. Exp. Gerontol. 124, 110653. 10.1016/j.exger.2019.110653 31295526

[B22] Iyaswamy A. Krishnamoorthi S. K. Liu Y. W. Song J. X. Kammala A. K. Sreenivasmurthy S. G. (2020a). Yuan-Hu Zhi Tong Prescription Mitigates Tau Pathology and Alleviates Memory Deficiency in the Preclinical Models of Alzheimer's disease. Front. Pharmacol. 11, 584770. 10.3389/fphar.2020.584770 33192524 PMC7663173

[B23] Iyaswamy A. Krishnamoorthi S. K. Song J.-X. Yang C.-B. Kaliyamoorthy V. Zhang H. (2020b). NeuroDefend, a Novel Chinese Medicine, Attenuates Amyloid-β and Tau Pathology in Experimental Alzheimer's Disease models. J. Food Drug Anal. 28, 132–146. 10.1016/j.jfda.2019.09.004 31883601 PMC13521053

[B24] Jin F. Wu Q. Lu Y.-F. Gong Q.-H. Shi J.-S. (2008). Neuroprotective Effect of Resveratrol on 6-OHDA-Induced Parkinson's disease in rats. Eur. J. Pharmacol. 600, 78–82. 10.1016/j.ejphar.2008.10.005 18940189

[B25] Jonsson G. (1980). Chemical Neurotoxins as Denervation Tools in neurobiology. Annu. Rev. Neurosci. 3, 169–187. 10.1146/annurev.ne.03.030180.001125 6106449

[B26] Khan M. M. Ahmad A. Ishrat T. Khan M. B. Hoda M. N. Khuwaja G. (2010). Resveratrol Attenuates 6-Hydroxydopamine-Induced Oxidative Damage and Dopamine Depletion in Rat Model of Parkinson's disease. Brain Res. 1328, 139–151. 10.1016/j.brainres.2010.02.031 20167206

[B27] Kraeuter A. K. Guest P. C. Sarnyai Z. (2019). The Y-Maze for Assessment of Spatial Working and Reference Memory in mice. Methods Mol Biol., 1916, 105–111. 10.1007/978-1-4939-8994-2_10 30535688

[B28] Lashuel H. A. Overk C. R. Oueslati A. Masliah E. (2013). The Many Faces of α-synuclein: from Structure and Toxicity to Therapeutic target. Nat. Rev. Neurosci. 14, 38–48. 10.1038/nrn3406 23254192 PMC4295774

[B29] Liberatore G. T. Jackson-Lewis V. Vukosavic S. Mandir A. S. Vila M. Mcauliffe W. G. (1999). Inducible Nitric Oxide Synthase Stimulates Dopaminergic Neurodegeneration in the MPTP Model of Parkinson disease. Nat. Med. 5, 1403–1409. 10.1038/70978 10581083

[B30] Lim S.-Y. Lang A. E. (2010). The Nonmotor Symptoms of Parkinson's Disease-An overview. Mov. Disord. 25, S123–S130. 10.1002/mds.22786 20187234

[B31] Liu L.-F. Song J.-X. Lu J.-H. Huang Y.-Y. Zeng Y. Chen L.-L. (2015). Tianma Gouteng Yin, a Traditional Chinese Medicine Decoction, Exerts Neuroprotective Effects in Animal and Cellular Models of Parkinson’s disease. Scientific Rep. 5, 16862. 10.1038/srep16862 PMC4649620 26578166

[B32] Liu Q. Zhu D. Jiang P. Tang X. Lang Q. Yu Q. (2019). Resveratrol Synergizes with Low Doses of L-DOPA to Improve MPTP-Induced Parkinson Disease in mice. Behav. Brain Res. 367, 10–18. 10.1016/j.bbr.2019.03.043 30922940

[B33] Lofrumento D. D. Nicolardi G. Cianciulli A. Nuccio F. D. Pesa V. L. Carofiglio V. (2014). Neuroprotective Effects of Resveratrol in an MPTP Mouse Model of Parkinson's-like Disease: Possible Role of SOCS-1 in Reducing Pro-inflammatory responses. Innate Immun. 20, 249–260. 10.1177/1753425913488429 23764428

[B34] Lu K.-T. Ko M.-C. Chen B.-Y. Huang J.-C. Hsieh C.-W. Lee M.-C. (2008). Neuroprotective Effects of Resveratrol on MPTP-Induced Neuron Loss Mediated by Free Radical scavenging. J. Agric. Food Chem. 56, 6910–6913. 10.1021/jf8007212 18616261

[B35] Lueptow L. M. (2017). Novel Object Recognition Test for the Investigation of Learning and Memory in mice. J Vis Exp., e55718. 10.3791/55718 PMC5614391 28892027

[B36] Mccormack A. L. Thiruchelvam M. Manning-Bog A. B. Thiffault C. Langston J. W. Cory-Slechta D. A. (2002). Environmental Risk Factors and Parkinson's Disease: Selective Degeneration of Nigral Dopaminergic Neurons Caused by the Herbicide paraquat. Neurobiol. Dis. 10, 119–127. 10.1006/nbdi.2002.0507 12127150

[B37] Meade R. M. Fairlie D. P. Mason J. M. (2019). Alpha-synuclein Structure and Parkinson’s Disease–Lessons and Emerging principles. Mol. neurodegeneration. 14, 1–14. 10.1186/s13024-019-0329-1 PMC6647174 31331359

[B38] Mudò G. Mäkelä J. Liberto V. D. Tselykh T. V. Olivieri M. Piepponen P. (2012). Transgenic Expression and Activation of PGC-1α Protect Dopaminergic Neurons in the MPTP Mouse Model of Parkinson's disease. Cell. Mol. Life Sci. 69, 1153–1165. 10.1007/s00018-011-0850-z 21984601 PMC11114858

[B39] Okawara M. Katsuk H. Kurimoto E. Shibata H. Kume T. Akaike A. (2007). Resveratrol Protects Dopaminergic Neurons in Midbrain Slice Culture from Multiple insults. Biochem. Pharmacol. 73, 550–560. 10.1016/j.bcp.2006.11.003 17147953

[B40] Oliveira A. Monteiro V. Navegantes-Lima K. Reis J. Gomes R. Rodrigues D. (2017). Resveratrol Role in Autoimmune Disease-A Mini-review. Nutrients. 9, 1306. 10.3390/nu9121306 PMC5748756 29194364

[B41] Palle S. Neerati P. (2018). Improved Neuroprotective Effect of Resveratrol Nanoparticles as Evinced by Abrogation of Rotenone-Induced Behavioral Deficits and Oxidative and Mitochondrial Dysfunctions in Rat Model of Parkinson's disease. Naunyn-schmiedeberg's Arch. Pharmacol. 391, 445–453. 10.1007/s00210-018-1474-8 29411055

[B42] Poewe W. Seppi K. Tanner C. M. Halliday G. M. Brundin P. Volkmann J. (2017). Parkinson disease. Nat. Rev. Dis. primers 3, 1–21. 10.1038/nrdp.2017.13 28332488

[B43] Prut L. Belzung C. (2003). The Open Field as a Paradigm to Measure the Effects of Drugs on Anxiety-like Behaviors: a review. Eur. J. Pharmacol. 463, 3–33. 10.1016/s0014-2999(03)01272-x 12600700

[B44] Pycock C. J. Carter C. J. Kerwin R. W. (1980). Effect of 6-Hydroxydopamine Lesions of the Medial Prefrontal Cortex on Neurotransmitter Systems in Subcortical Sites in the rat. J. Neurochem. 34, 91–99. 10.1111/j.1471-4159.1980.tb04625.x 6161214

[B45] Schober A. (2004). Classic Toxin-Induced Animal Models of Parkinson?s Disease: 6-OHDA and MPTP. Cell Tissue Res 318, 215–224. 10.1007/s00441-004-0938-y 15503155

[B46] Sherer T. B. Betarbet R. Testa C. M. Seo B. B. Richardson J. R. Kim J. H. (2003). Mechanism of Toxicity in Rotenone Models of Parkinson's disease. J. Neurosci. 23, 10756–10764. 10.1523/jneurosci.23-34-10756.2003 14645467 PMC6740985

[B47] Shiotsuki H. Yoshimi K. Shimo Y. Funayama M. Takamatsu Y. Ikeda K. (2010). A Rotarod Test for Evaluation of Motor Skill learning. J. Neurosci. Methods 189, 180–185. 10.1016/j.jneumeth.2010.03.026 20359499

[B48] Srivastava G. Dixit A. Yadav S. Patel D. K. Prakash O. Singh M. P. (2012). Resveratrol Potentiates Cytochrome P450 2d22-Mediated Neuroprotection in Maneb- and Paraquat-Induced Parkinsonism in the mouse. Free Radic. Biol. Med. 52, 1294–1306. 10.1016/j.freeradbiomed.2012.02.005 22334051

[B49] Tanner C. M. Kamel F. Ross G. W. Hoppin J. A. Goldman S. M. Korell M. (2011). Rotenone, Paraquat, and Parkinson's disease. Environ. Health Perspect 119, 866–872. 10.1289/ehp.1002839 21269927 PMC3114824

[B50] Wang Y. Xu H. Fu Q. Ma R. Xiang J. (2011). Protective Effect of Resveratrol Derived from Polygonum Cuspidatum and its Liposomal Form on Nigral Cells in Parkinsonian rats. J. Neurol. Sci. 304, 29–34. 10.1016/j.jns.2011.02.025 21376343

[B51] Wang Z.-H. Zhang J.-L. Duan Y.-L. Zhang Q.-S. Li G.-F. Zheng D.-L. (2015). MicroRNA-214 Participates in the Neuroprotective Effect of Resveratrol via Inhibiting α-synuclein Expression in MPTP-Induced Parkinson's Disease mouse. Biomed. Pharmacother. 74, 252–256. 10.1016/j.biopha.2015.08.025 26349993

[B52] Wang Z.-Y. Liu J.-Y. Yang C.-B. Malampati S. Huang Y.-Y. Li M.-X. (2017). Neuroprotective Natural Products for the Treatment of Parkinson's Disease by Targeting the Autophagy-Lysosome Pathway: A Systematic review. Phytother. Res. 31, 1119–1127. 10.1002/ptr.5834 28504367

[B53] Wang Z. Y. Liu J. Zhu Z. Su C. F. Sreenivasmurthy S. G. Iyaswamy A. (2020). Traditional Chinese Medicine Compounds Regulate Autophagy for Treating Neurodegenerative Disease: A Mechanism review. Biomed. Pharmacother. 133, 110968. 10.1016/j.biopha.2020.110968 33189067

[B54] Widdowson P. Farnworth M. Simpson M. Lock E. (1996). Influence of Age on the Passage of Paraquat through the Blood-Brain Barrier in Rats: a Distribution and Pathological examination. Hum. Exp. Toxicol. 15, 231–236. 10.1177/096032719601500308 8839211

[B55] Xia D. Sui R. Zhang Z. (2019). Administration of Resveratrol Improved Parkinson's Disease‐like Phenotype by Suppressing Apoptosis of Neurons via Modulating the MALAT1/miR‐129/SNCA Signaling pathway. J. Cel Biochem. 120, 4942–4951. 10.1002/jcb.27769 30260025

[B56] Zhang L.-F. Yu X.-L. Ji M. Liu S.-Y. Wu X.-L. Wang Y.-J. (2018). Resveratrol Alleviates Motor and Cognitive Deficits and Neuropathology in the A53T α-synuclein Mouse Model of Parkinson's disease. Food Funct. 9, 6414–6426. 10.1039/c8fo00964c 30462117

[B57] Zhao X. Wang J. Hu S. Wang R. Mao Y. Xie J. (2017). Neuroprotective Effect of Resveratrol on Rotenone-Treated C57BL/6 mice. Neuroreport 28, 498–505. 10.1097/wnr.0000000000000789 28471847

[B58] Zhuang X.-X. Wang S.-F. Tan Y. Song J.-X. Zhu Z. Wang Z.-Y. (2020). Pharmacological Enhancement of TFEB-Mediated Autophagy Alleviated Neuronal Death in Oxidative Stress-Induced Parkinson’s Disease Models. Cel Death Dis. 11, 1–18. 10.1038/s41419-020-2322-6 PMC7028954 32071296

